# Impact of Ethanol Stress on *Yarrowia lipolytica* for Sustainable Bioconversion of Agro-Food Oil Wastes into Lipases and Lipids

**DOI:** 10.3390/foods14213696

**Published:** 2025-10-29

**Authors:** Amina Laribi, Joanna Bryś, Abderrahmane Selmania, Assia Ikhlef, Insaf Btaïche, Abdelghani Mouzai, Bartłomiej Zieniuk, Doria Naila Bouchedja

**Affiliations:** 1 Laboratory of Food Sciences, Formulation, Innovation, Valorization & Artificial Intelligence (SAFIVIA), Biomass Production Modeling & Enzyme Valorization Team, Institute of Nutrition and Agri-Food Technologies (INATAA), University of Constantine 1—Frères Mentouri (UFMC1), Route de Aïn El Bey, Constantine 25000, Algeria; amina.laribi@doc.umc.edu.dz; 2 Department of Chemistry, Institute of Food Sciences, Warsaw University of Life Sciences-SGGW, 159C Nowoursynowska Str., 02-776 Warsaw, Poland; joanna_brys@sggw.edu.pl (J.B.); bartlomiej_zieniuk@sggw.edu.pl (B.Z.); 3 Biotechnology Research Center (CRBt), Nouvelle Ville Ali Mendjeli, Constantine 25000, Algeria; a.selmania@crbt.dz (A.S.); a.ikhlef@crbt.dz (A.I.); 4 Laboratory of Mycology, Biotechnology and Microbial Activity, Faculty of Natural and Life Sciences, University of Constantine 1—Frères Mentouri (UFMC1), Route de Aïn El Bey, Constantine 25000, Algeria; bataiche.insaf@umc.edu.dz; 5 Laboratory of Agri-Food Engineering, Food Process Engineering (GENIAL), Biodiversity and Agro-Environment Team, University of Constantine 1—Frères Mentouri (UFMC1), Route de Aïn El Bey, Constantine 25000, Algeria; abdo_agroalimentaire@yahoo.fr

**Keywords:** hydrophobic substrates, oxidative stability, fatty acid remodeling, lipid metabolism adaptation, biocatalyst development, sustainable biorefinery

## Abstract

Ethanol stress profoundly affects yeast metabolism, yet its integrated impact on lipase activity and lipid remodeling in *Yarrowia lipolytica* remains unexplored. Here, we investigated, for the first time, the combined effects of ethanol-induced stress on lipase production and fatty acid profiles in *Y. lipolytica* cultivated on two hydrophobic substrates: olive mill wastewater (OMW) and Waste Frying Oil (WFO). Ethanol was applied at increasing concentrations (3%, 5%, and 7% *v*/*v*), and the physiological responses were monitored over time (48, 72, and 96 h). Our results reveal a substrate-dependent and dose-dependent response to ethanol. Lipase activity was significantly enhanced at 5% ethanol, reaching 0.55 ± 0.11 U/mL in the OMW medium after 48 h. In comparison, mild stress (3%) induced the de novo synthesis of C20:1 (eicosenoic acid) and C20:2 (eicosadienoic acid), indicating reprogramming of lipid biosynthetic pathways. Oxidative stability, assessed by pressurized differential scanning calorimetry (PDSC), markedly improved in OMW-derived lipids, with τ_on_ increasing from 30.48 ± 0.80 to 47.07 ± 3.92 min and τ_max_ from 35.73 ± 0.62 to 54.04 ± 1.99 min under 3% ethanol. Conversely, WFO-derived samples exhibited lower oxidative stability and less pronounced changes in lipid composition. These findings demonstrate that *Y. lipolytica* adapts its lipid metabolism differently depending on the substrate, and that controlled ethanol exposure can enhance both lipase secretion and lipid oxidative resistance, underscoring its potential as a robust biocatalyst for sustainable biorefineries and the valorization of agro-food oil wastes.

## 1. Introduction

Oleaginous yeasts are strategic microorganisms in biotechnology due to their ability to accumulate significant amounts of lipids and produce industrially relevant enzymes such as lipases [1]. Among them, *Yarrowia lipolytica* stands out for its remarkable metabolic flexibility, enabling it to valorize a wide variety of substrates, including agro-industrial wastes such as olive mill wastewater and used cooking oils [2,3,4]. This capability offers a dual advantage: fostering a circular bioeconomy by converting carbon-rich effluents into high-value biomolecules while reducing enzyme production costs [5,6].

Lipases (EC 3.1.1.3), hydrolase enzymes that catalyze the stepwise hydrolysis of triacylglycerols—yielding diacylglycerols, monoacylglycerols, free fatty acids, and glycerol—play a central role in numerous industrial processes, ranging from biodiesel production to the formulation of food, pharmaceutical, and cosmetic products [7]. Their robustness under extreme conditions and broad catalytic versatility make *Y. lipolytica* lipases highly attractive biocatalysts for innovative applications [3]. In parallel, microbial oils enriched in fatty acids produced by this yeast are increasingly recognized as sustainable alternatives to plant-derived oils, particularly in the context of transitioning towards renewable resources [8,9].

However, the biosynthesis of lipids and lipases in *Y. lipolytica* is highly sensitive to environmental cues and stressors [8]. Ethanol stress, in particular, poses a significant challenge by compromising membrane integrity, perturbing lipid bilayer fluidity, and triggering oxidative damage through the excessive accumulation of reactive oxygen species (ROS) [10]. Such stress not only affects the physical properties of cellular membranes but also disrupts key metabolic pathways involved in energy production and macromolecule synthesis. To counteract these effects, yeasts activate intricate adaptive mechanisms, including antioxidant defense systems, remodeling of membrane lipid composition, and global metabolic reprogramming to restore redox balance and cellular homeostasis. In *Y. lipolytica*, ethanol-induced stress triggers a multifaceted adaptive response involving activation of antioxidant systems and reorganization of central metabolism to maintain redox equilibrium. Similarly, in other oleaginous yeasts such as *Pichia pastoris* and *Rhodotorula toruloides*, alcohol stress has been shown to markedly influence lipidomic and proteomic profiles, thereby modulating enzyme synthesis and lipid composition [10,11,12]. Furthermore, a recent study highlighted the emergence of alcohol dehydrogenase (ADH) activity in *Y. lipolytica* under various stress conditions, in correlation with the activation of antioxidant enzymes such as catalase and superoxide dismutase. This finding suggests the existence of tightly linked metabolic mechanisms between oxidative response and ethanol metabolism [10].

We hypothesize that moderate ethanol stress triggers coordinated physiological and metabolic responses in *Y. lipolytica*. These responses lead to changes in cell shape, regulate extracellular lipase production, and alter lipid profile dynamics—adaptations aimed at maintaining cell viability and productivity under stress. Our study focused on four main goals: (i) measuring how ethanol affects cell morphology and growth; (ii) examining the impact of various ethanol levels on lipase secretion; (iii) analyzing changes in lipid profiles to understand underlying metabolic adjustments; and (iv) comparing these responses when the yeast is grown on two different carbon sources—used frying oil and olive mill wastewater. To our knowledge, this is the first comprehensive study on how ethanol stress influences lipase activity and lipid metabolism in *Y. lipolytica* cultivated on residual agro-industrial substrates. The findings support efforts in metabolic engineering and the sustainable reuse of carbon-rich waste products.

## 2. Materials and Methods

### 2.1. Microbial Strain, Materials, and Culture Conditions

The wild-type yeast strain *Y. lipolytica* L2 (GenBank accession number: KF156787) employed in this work originated from the Laboratory of Biotechnology and Food Quality (BIOCAL). It was initially isolated from raw milk samples at the Institute of Nutrition, Food, and Agro-Food Technologies (INATAA) at the University Frères Mentouri—Constantine 1 (Algeria). For long-term conservation, the strain was preserved on YPD medium containing 30% (*v*/*v*) sterile glycerol and stored at −20 °C in cryovials until required.

To reactivate the culture, frozen stocks were streaked onto solid YPD agar plates composed of 10 g/L meat extract, 5 g/L yeast extract, 15 g/L glucose, and 18 g/L agar. Plates were incubated at 29 ± 1 °C for 48–72 h to allow colony formation. A single colony was then used to inoculate 100 mL of liquid YPD medium (10 g/L casein peptone, 5 g/L yeast extract, 20 g/L glucose) in 250-mL Erlenmeyer flasks. The pre-culture was incubated at 29 ± 1 °C with shaking (185 rpm) for 24 h to obtain actively growing cells in the exponential phase [8,13].

For the main experiments, cells from the pre-culture were transferred into YP medium (20 g/L peptone, 10 g/L yeast extract) to reach an initial optical density (OD_600_) of 0.1. Two different hydrophobic substrates were used as carbon sources, each added at a final concentration of 200 g/L: used cooking oil (WCO), collected from restaurants in Warsaw (Poland), and olive mill wastewater (OMW), obtained in May 2024 from an olive oil extraction facility in Béjaïa, Algeria, with a polyphenol concentration of 2 g/L. Cultures were carried out in 250-mL Erlenmeyer flasks containing 100 mL of medium, incubated at 29 ± 1 °C, and agitated at 180 rpm [5] in an IKA KS 4000 ic control shaker (IKA company, Konigswinter, Germany).

All chemical reagents and solvents used in this study were obtained from Sigma-Aldrich (Poznań, Poland) and Avantor Performance Materials Poland S.A. (Gliwice, Poland). The microbiological media components were purchased from BTL (Łódź, Poland).

### 2.2. Ethanol Stress Induction

Ethanol stress was applied to *Y. lipolytica* cultures 24 h after inoculation, regardless of the growth phase. To prevent abrupt metabolic inhibition, ethanol was introduced progressively over 1–2 h, allowing cells to adapt gradually to the changing environment. Four experimental conditions were established: a control culture without ethanol and three treatments containing 3%, 5%, and 7% (*v*/*v*) ethanol. Each condition was tested in triplicate independent cultures, following a protocol modified from previously reported methods [14,15].

To evaluate the cellular response to ethanol exposure, several analyses were conducted at 24-h intervals. Biomass accumulation was quantified as dry cell weight, and microscopic examinations were performed to identify morphological changes, such as cellular aggregation or deformation, associated with ethanol stress. Lipase activity in the extracellular medium was determined enzymatically, and the harvested biomass was used for microbial oil extraction and fatty acid profiling to assess the effects of ethanol stress on lipid metabolism and biosynthesis.

### 2.3. Determination of Dry Cell Weight (DCW)

Cultures were carried out in 250 mL Erlenmeyer flasks containing 100 mL of culture medium. Following ethanol stress induction, 10 mL samples were collected every 24 h, corresponding to mid-exponential, late-exponential, and stationary growth phases, to monitor cell growth kinetics. Cells were harvested by centrifugation at 8000× *g* for 10 min at 4 °C using a SIGMA Laborzentrifugen D-37520 centrifuge (Sigma, Osterode am Harz, Germany). The resulting supernatant was retained for subsequent analysis of extracellular lipase activity.

The cell pellets were washed twice with distilled water to remove any extracellular lipids that might have been adsorbed onto the cell surface. Each wash was followed by centrifugation under the same conditions.

The biomass was then dried in an oven at 80 °C overnight and weighed to determine the dry cell weight (DCW, expressed in g·L^−1^) by gravimetric analysis. Reported values represent the average of three independent replicates [16].

### 2.4. Lipase Activity Assay

The crude lipase preparation was obtained after centrifugation at 8000 rpm for 10 min to remove cells and debris. Enzymatic activity was determined by spectrophotometry using the hydrolysis of *p*-nitrophenyl laurate at 37 °C. For each assay, 100 μL of the supernatant was mixed with 25 μL of a *p*-nitrophenyl laurate solution (0.3 mmol dissolved in 2 mL of heptane) in Eppendorf tubes. The reaction mixture was incubated for 15 min at 37 °C, and the absorbance was immediately measured at 410 nm using a UV-Vis spectrophotometer (Rayleigh UV-1601, BRAIC, Beijing, China) [17].

One unit (U) of lipase activity corresponds to the amount of enzyme that liberates 1 μmol of *p*-nitrophenol per minute under the described conditions.

### 2.5. Determination of Fatty Acid Composition

Total lipids were extracted from freeze-dried yeast cells that had been finely ground with a mortar and pestle. The extraction process followed the Soxhlet method, employing n-hexane as the extraction solvent. After completion, the solvent was evaporated, and the intracellular lipid content was quantified.

The fatty acid (FA) profile was determined through gas chromatography (GC) analysis of fatty acid methyl esters (FAMEs). Methyl esters were prepared from the extracted lipids by transesterification with sodium methanolate, according to ISO 5509:2001 [18]. Gas chromatography was employed owing to its sensitivity, precision, and ability to resolve complex fatty acid mixtures.

Chromatographic analyses were carried out using a YL6100 gas chromatograph (Young Lin Bldg., Anyang, Hogye-dong, South Korea) equipped with a flame ionization detector (FID) and a BPX-70 capillary column (60 m × 0.25 mm i.d., 0.25 µm film thickness, SGE Analytical Science, Milton Keynes, UK). The oven temperature program began at 70 °C for 30 s, increased at 15 °C/min to 160 °C, then at 1.1 °C/min to 200 °C (maintained for 12 min), and finally at 30 °C/min to 225 °C, held for 1 min. Injector and detector temperatures were set to 225 °C and 250 °C, respectively, with a split ratio of 1:50. Nitrogen was used as the carrier gas at a constant flow rate of 1 mL/min.

Fatty acid composition was expressed as relative percentages derived from peak areas. Identification of each fatty acid was achieved by comparing the retention times of the sample FAME peaks with those of a standard Supelco 37 Component FAME Mix [19].

### 2.6. Oxidative Stability Determination

Oxidative stability of the obtained microbial oils was assessed using pressure differential scanning calorimetry (PDSC) with a DSC Q20 TA instrument (TA Instruments, New Castle, DE, USA) equipped with a high-pressure cell. Fat samples (3–4 mg) were placed in open aluminum pans in a cell with an empty reference pan and subjected to an initial oxygen pressure of 1400 kPa. The analysis was conducted under isothermal conditions at 120 °C. Data were processed using the TA Universal Analysis 2000 software (TA Instruments, New Castle, DE, USA). The induction time (τ_on_) and the maximum oxidation time (τ_max_), were determined [20].

### 2.7. Statistical Analysis

Data were analyzed using Statistica 13.3 (TIBCO Software Inc., Palo Alto, CA, USA). The study employed a one-factor design with treatment (ethanol concentration) as the independent variable. Group means were compared through one-way ANOVA (α = 0.05) followed by Tukey’s HSD post hoc test. Assumption checks were performed on model residuals using the Shapiro–Wilk test for normality and Levene’s test for homogeneity. Unless indicated otherwise, data are presented as the mean of triplicates with standard deviation (SD).

## 3. Results

This study aimed to evaluate the impact of ethanol-induced stress on growth, cell viability, extracellular lipase activity, and lipid biosynthesis in the strain *Y. lipolytica* L2 (KF156787), cultivated on two distinct hydrophobic substrates: waste frying oil (WFO) and olive mill wastewater (OMW). The objective was also to compare the respective effects of ethanol stress and carbon source type on the physiological and metabolic responses of the yeast. To achieve this, a series of analyses was performed at different incubation times in the presence of increasing ethanol concentrations (3%, 5%, and 7% *v*/*v*). The results obtained are presented and discussed below with reference to literature data in order to better understand the adaptive mechanisms deployed by *Y. lipolytica* under ethanol-induced oxidative stress, depending on the nature of the substrate used.

### 3.1. Dry Cell Weight (DCW) and Intracellular Lipid Yields

**Figure 1:**
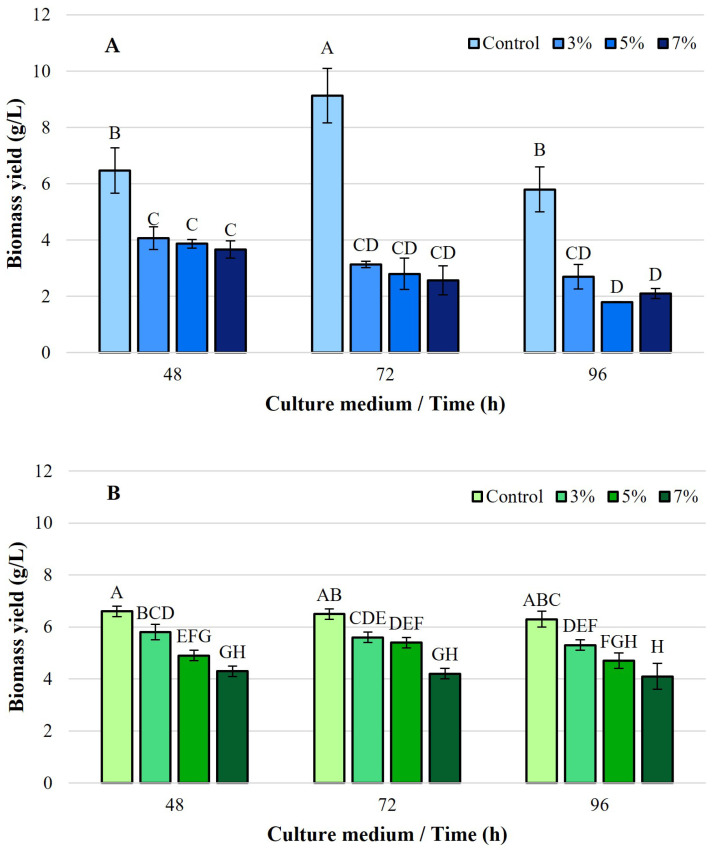
Biomass yield (g/L) of *Y. lipolytica* L2 (KF156787) under ethanol stress at 48, 72, and 96 h. (**A**) Medium with waste frying oil (WFO) as the carbon source; (**B**) medium with olive mill wastewater (OMW) as the carbon source. Bars represent Control (0% ethanol), 3%, 5%, and 7% (*v*/*v*) ethanol. Error bars represent the standard deviation (SD) of three independent measurements. The values with the same uppercase letters (A–H) did not differ significantly (Tukey’s HSD test, α = 0.05).

The growth of *Y. lipolytica* L2 dry cell biomass was monitored over 96 h under both control and ethanol-induced stress conditions (3%, 5%, and 7% *v*/*v*), using either spent cooking oil or olive mill effluent as the sole carbon source (Figure 1).

In ethanol-free control cultures, efficient biomass development was observed in both media. When grown on WFO, biomass increased from 6.47 ± 0.81 g/L at 48 h to a maximum of 9.13 ± 0.97 g/L at 72 h, followed by a decline to 5.80 ± 0.80 g/L at 96 h. This pattern suggests an active growth phase followed by a slowdown in cellular activity, possibly due to nutrient depletion or accumulation of inhibitory by-products. In contrast, cultures grown on OMW displayed more stable biomass values throughout the incubation period, ranging between 6.60 ± 0.20 g/L at 48 h and 6.30 ± 0.30 g/L at 96 h, indicating a more moderate but continuous growth dynamic.

The addition of ethanol to the culture media resulted in a clear, dose-dependent inhibition of *Y. lipolytica* L2 growth. In the presence of 3% ethanol, biomass in the WFO medium decreased from 4.07 ± 0.40 g/L at 48 h to 2.70 ± 0.44 g/L at 96 h. This reduction was more pronounced at 5% ethanol, where biomass decreased from 3.87 ± 0.15 g at 48 h to 1.80 ± 0.01 g/L at 96 h. At 7%, growth was severely impaired, with biomass dropping from 3.67 ± 0.31 g/L at 48 h to 2.10 ± 0.17 g/L at 96 h.

A similar trend was observed in the OMW-based media, although biomass levels were slightly higher than those recorded with waste oil at the same ethanol concentrations. In 3% ethanol cultures, biomass decreased from 5.80 ± 0.30 g/L at 48 h to 5.30 ± 0.20 g/L at 96 h. At 5% ethanol, biomass declined from 4.90 ± 0.20 g/L to 4.70 ± 0.30 g/L, while at 7% ethanol, it decreased from 4.30 ± 0.20 g/L to 4.10 ± 0.50 g/L over the 96 h incubation. Overall, these findings indicate a progressive inhibition of cell growth with increasing ethanol concentrations, regardless of the carbon source used. However, growth appeared to be slightly better sustained in OMW-containing media, although overall biomass levels remained lower than those obtained with waste cooking oil under control (ethanol-free) conditions.

**Figure 2:**
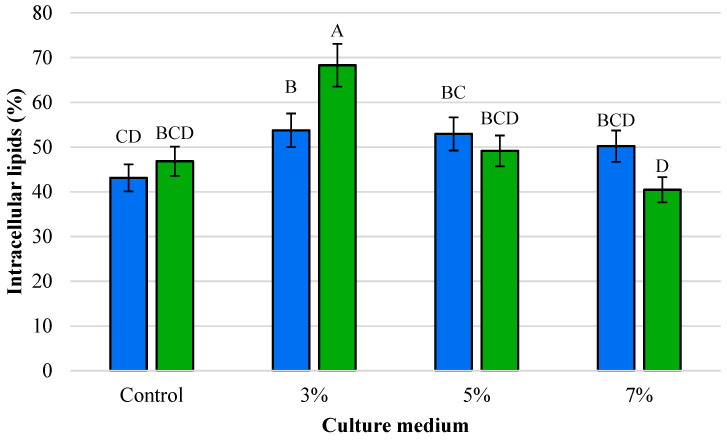
Intracellular lipids (%) of *Y. lipolytica* L2 (KF156787) under ethanol stress in two media. Blue bars: medium with waste frying oil (WFO); green bars: medium with olive mill wastewater (OMW). Treatments: Control (0% ethanol), 3%, 5%, and 7% (*v*/*v*). The values with the same uppercase letters (A–D) did not differ significantly (Tukey’s HSD test, α = 0.05).

The intracellular lipid content of *Y. lipolytica* L2 was determined after 96 h of cultivation in both waste frying oil (WFO) and olive mill wastewater (OMW) media under control and ethanol-induced stress conditions (3%, 5%, and 7% *v*/*v* ethanol). In control cultures without ethanol, lipid accumulation reached 43.13% in WFO medium and 46.82% in OMW medium. These values confirm the oleaginous nature of *Y. lipolytica* and highlight that both carbon sources supported efficient lipid biosynthesis (Figure 2).

Ethanol supplementation had a marked influence on intracellular lipid yields. In WFO medium, the presence of 3% ethanol promoted lipid accumulation, resulting in a maximum yield of 53.74%, representing a significant increase compared to the control. At 5% ethanol, lipid content remained elevated (52.94%), whereas at 7% ethanol, yields declined to 50.19%, indicating partial inhibition of lipid biosynthesis under higher ethanol stress. A similar trend was observed in OMW medium, but with more pronounced fluctuations. At 3% ethanol, the lipid content increased sharply to 68.28%, representing the highest yield across all tested conditions. However, at 5% ethanol, yields dropped to 49.14%, close to the control level, and further declined to 40.45% at 7% ethanol. This suggests that while low ethanol levels can act as a metabolic stressor, enhancing lipid accumulation, higher ethanol concentrations exert an inhibitory effect. Overall, the results demonstrate that ethanol supplementation can stimulate intracellular lipid biosynthesis at moderate concentrations (3–5%), especially in OMW-based cultures. However, excessive ethanol stress (7%) reduces lipid accumulation in both media, reflecting the sensitivity of *Y. lipolytica* lipid metabolism to environmental stressors. Ethanol supplementation significantly affected intracellular lipid yields.

### 3.2. Morphological Observation of Yeast Under Light Microscopy

**Figure 3:**
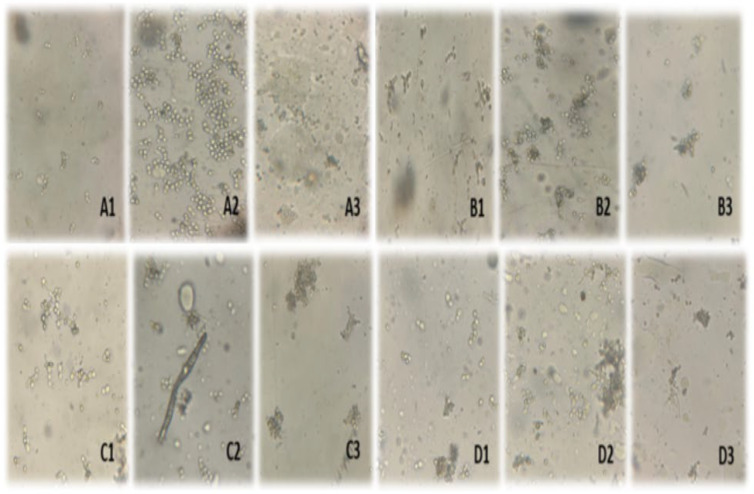
Microscopic observation of *Y. lipolytica* L2 (KF156787) cells cultured for 48 h, 72 h, and 96 h under varying ethanol concentrations (0%, 3%, 5%, and 7% *v*/*v*). (**A**) control without ethanol; (**B**) 3% ethanol; (**C**) 5% ethanol; (**D**) 7% ethanol; Sub-panels: 1: after 48 h of cultivation/24 h of ethanol exposure; 2—after 72 h of cultivation/48 h of ethanol exposure; 3: after 96 h of cultivation/72 h of ethanol exposure.

Microscopic examination of *Y. lipolytica* L2 (KF156787) revealed a progressive morphological response to ethanol-induced stress (Figure 3). In the absence of ethanol, cells exhibited a typical ovoid morphology, appearing isolated and evenly distributed across the microscopic field, with no signs of aggregation or deformation, regardless of the carbon source used.

At 3% ethanol, slight cell aggregation appeared after 48 h and gradually increased by 72–96 h, while most cells preserved their normal shape. At 5%, aggregation became extensive, and filamentous forms emerged, particularly after 72 h in OMW-based medium. At 7%, severe morphological alterations were observed from 48 h onward, including irregular clumps, loss of ovoid shape, and partial cell disintegration at later stages. Quantitatively, about 35% of cells formed small aggregates at 3% ethanol after 48 h, increasing to approximately 58% at 5%, with filamentous structures becoming evident after 72 h. At 7%, nearly 72% of cells displayed aggregation or filamentous morphology, accompanied by advanced deformation and fragmentation, regardless of the substrate used.

### 3.3. Lipase Activity

**Figure 4:**
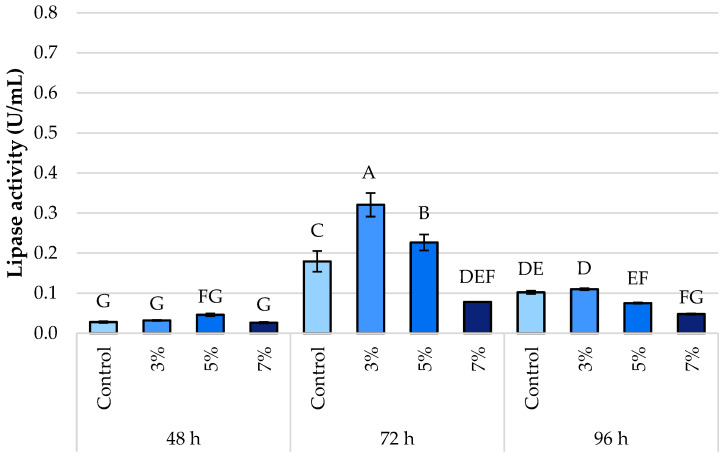
Lipase activity of *Y. lipolytica* L2 (KF156787) in medium with waste frying oil under control conditions and in response to ethanol stress (3%, 5%, and 7% *v*/*v*). Error bars represent the standard deviation (SD) of three independent measurements. The values with the same uppercase letters (A–G) did not differ significantly (Tukey’s HSD test, α = 0.05).

**Figure 5:**
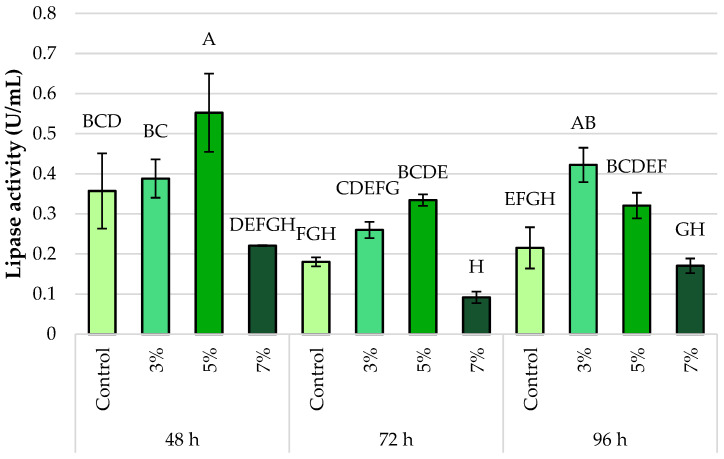
Lipase activity of *Y. lipolytica* L2 (KF156787) in olive mill wastewater medium under control conditions and in response to ethanol stress (3%, 5%, and 7% *v*/*v*). Error bars represent the standard deviation (SD) of three independent measurements. The values with the same uppercase letters (A–H) did not differ significantly (Tukey’s HSD test, α = 0.05).

The evolution of lipase activity in *Y. lipolytica* L2 was also evaluated in two different culture media: one supplemented with waste frying oil (Figure 4) and the other with OMW (Figure 5), in the presence of varying ethanol concentrations (0%, 3%, 5%, and 7%, *v*/*v*) and at three incubation times (48, 72, and 96 h).

After 48 h of incubation, the highest lipase activity was recorded in the OMW-based medium supplemented with 5% ethanol, reaching 0.55 ± 0.11 U/mL. In contrast, in the medium containing waste oil, enzymatic activity remained consistently low across all ethanol concentrations, not exceeding 0.05 U/mL. These findings suggest a more rapid induction of lipase expression in the OMW medium, likely due to improved accessibility of lipidic substrates.

After 72 h of incubation, lipase activity increased significantly in the waste oil medium, reaching a peak at 3% ethanol (0.32 ± 0.01 U/mL). In contrast, activity in the OMW-based medium showed a slight decline, remaining at moderate levels (ranging from 0.29 to 0.34 U/mL, depending on the ethanol concentration). However, a considerable decrease in activity to 0.08 ± 0.01 U/mL—the lowest recorded value in this group—is observed, indicating a severe inhibition at 7% ethanol.

After 96 h, the different dynamics of the two environments become apparent. At 3% ethanol (0.44 ± 0.05 U/mL), lipase activity remains high in the OMW-based medium. In contrast, a slight reduction in activity is observed under all tested conditions in the WFO-based medium, with values ranging from 0.09 ± 0.01 to 0.05 ± 0.01 U/mL. In the presence of OMW, this pattern suggests either enhanced enzymatic stability or prolonged lipase expression.

Finally, 7% ethanol markedly reduces lipase activity under both conditions, regardless of incubation time. Under these conditions, activity levels remain low, with values dropping below 0.20 U/mL in the OMW-based medium and below 0.10 U/mL in the waste oil-based medium.

### 3.4. Fatty Acid Composition of Yeast Lipids

The fatty acid profiles of waste frying oil (WFO) and olive mill wastewater (OMW) are shown in Table 1. Both substrates displayed typical long-chain fatty acid compositions, mainly C16 and C18 species. In OMW, oleic acid (C18:1) was the main component, making up about 43.7 ± 0.25% of total fatty acids, followed by linoleic acid (C18:2, 33.35 ± 0.37%) and palmitic acid (C16:0, 13.83 ± 0.71%). Minor components included stearic acid (C18:0, 3.95 ± 0.06%), α-linolenic acid (C18:3, 4.22 ± 0.10%), and palmitoleic acid (C16:1, 0.97 ± 0.05%). The total saturated fatty acids (ΣSFA) accounted for 17.78 ± 0.66%, while monounsaturated (ΣMUFA) and polyunsaturated fatty acids (ΣPUFA) represented 44.66 ± 0.20% and 37.57 ± 0.47%, respectively. In contrast, WFO showed a higher level of saturation and monounsaturation, with oleic acid (C18:1) remaining the dominant fatty acid (50.45 ± 2.43%), followed by linoleic acid (C18:2, 19.99 ± 4.04%) and stearic acid (C18:0, 14.44 ± 1.61%). Palmitic acid (C16:0) made up 11.71 ± 0.54%, while α-linolenic acid (C18:3) and palmitoleic acid (C16:1) were found in lower amounts (3.42 ± 0.54% and 0.00 ± 0.00%, respectively). The total SFA, MUFA, and PUFA contents were 26.15 ± 1.07%, 50.45 ± 2.43%, and 23.41 ± 3.50%, respectively. Overall, both OMW and WFO were characterized by a predominance of oleic acid (C18:1), although they differed significantly in their relative amounts of saturated and polyunsaturated fatty acids.

In the medium containing WFO (Table 2), a clear shift in fatty acid composition was observed under ethanol stress. Oleic acid (C18:1) increased substantially from 38.67 ± 1.59% in the control to 61.49 ± 0.19% at 7% ethanol, accompanied by a parallel rise in linoleic acid (C18:2). Minor fatty acids, including eicosenoic acid (C20:1), disappeared at ethanol concentrations ≥5%. Unlike the OMW-based medium, the total proportion of saturated fatty acids (SFA) remained relatively stable, ranging between 7.74% and 8.77%. Both monounsaturated fatty acids (MUFA) and polyunsaturated fatty acids (PUFA) progressively increased with ethanol supplementation.

In the OMW-based medium (Table 3), a progressive increase in oleic acid (C18:1) was also detected, rising from 40.56 ± 1.44% in the control to 48.61 ± 0.20% at 7% ethanol. In parallel, palmitoleic acid (C16:1) and palmitic acid (C16:0) decreased. Interestingly, rare fatty acids such as eicosenoic acid (C20:1) and eicosadienoic acid (C20:2) appeared at 3% ethanol but were no longer detectable at higher concentrations. The total SFA content significantly declined from 20.04 ± 2.11% to 13.08 ± 0.21%, suggesting a membrane adaptation mechanism to maintain lipid fluidity. MUFA levels increased steadily, whereas PUFA remained relatively stable throughout the ethanol treatments.

### 3.5. Oxidative Stability of Microbial Oils by Pressurized Differential Scanning Calorimetry (PDSC)

In the medium using WFO as a carbon source (Table 4), the microbial oils exhibited relatively low oxidative stabilities under all tested conditions. In the absence of ethanol (0%), the onset (τ_on_) and maximum (τ_max_) oxidation times were 1.02 ± 0.18 min and 3.45 ± 0.13 min, respectively. The addition of 3% ethanol slightly modified these parameters, with τ_on_ at 0.86 ± 0.45 min and τ_max_ at 5.02 ± 0.65 min. At 5% ethanol, τ_on_ further decreased to 0.50 ± 0.18 min and τ_max_ to 4.51 ± 0.37 min. At 7% ethanol, the values remained relatively stable, with τ_on_ at 0.97 ± 0.23 min and τ_max_ at 3.35 ± 0.67 min.

In the medium containing OMW as a carbon source (Table 5), microbial oils displayed more pronounced thermal variations depending on ethanol concentration. Under control conditions (0%), τ_on_ and τ_max_ were 30.48 ± 0.80 min and 35.73 ± 0.62 min, respectively. At 3% ethanol, both values increased significantly to 47.07 ± 3.92 min and 54.04 ± 1.99 min. However, at higher ethanol concentrations (5% and 7%), a sharp decline in thermal stability was recorded. At 5% ethanol, τ_on_ and τ_max_ dropped to 12.15 ± 0.12 min and 16.92 ± 0.16 min, while at 7% ethanol they reached their lowest values of 4.99 ± 0.21 min and 9.10 ± 0.37 min, respectively.

## 4. Discussion

### 4.1. Effect of Ethanol on Growth and Lipogenesis

The results clearly indicate a significant inhibitory effect of ethanol on the growth of *Y. lipolytica* L2 (KF156787), with the degree of inhibition increasing proportionally with ethanol concentration, irrespective of the carbon substrate used. However, this inhibitory effect was more pronounced in media containing olive mill wastewater, likely due to the presence of naturally occurring compounds with known antimicrobial activity.

At the cellular level, ethanol functions as a multifactorial stressor. By integrating into the lipid bilayer, it alters membrane fluidity and permeability, resulting in ion leakage, disruption of transport processes, and loss of cellular homeostasis. In response, yeasts often remodel their membrane lipid composition—adjusting the ratio of saturated to unsaturated fatty acids and modifying sterol content—to preserve structural integrity under ethanol-induced stress [21]. Ethanol exposure also intensifies oxidative stress by impairing mitochondrial function and stimulating the production of reactive oxygen species (ROS), which damage lipids, proteins, and nucleic acids. To counteract these effects, cells activate antioxidant defense systems, including superoxide dismutase, catalase, and glutathione, as well as heat shock proteins that contribute to restoring redox balance and maintaining protein stability [22]. At the metabolic level, ethanol disrupts key energy and carbon fluxes, including glycolysis, the tricarboxylic acid (TCA) cycle, and respiration, thereby limiting the availability of reducing cofactors (NADH and NADPH) essential for lipid biosynthesis. In *Y. lipolytica*, the induction of genes encoding alcohol dehydrogenases (ADH) and acetyl-CoA synthetase (ACS) suggests adaptive mechanisms for ethanol detoxification and potential assimilation [23].

Ethanol functions as a major cellular stressor by disrupting membrane integrity, interfering with key metabolic processes, and inducing oxidative stress. These combined effects compromise the overall metabolic performance and viability of the yeast cells. These observations are consistent with findings reported in the literature. For example, Yen et al. demonstrated that an ethanol concentration of approximately 9% almost completely inhibited the growth of non-conventional yeasts such as *Pichia anomala*. Similarly, Chandler et al. reported a significant reduction in the growth rate of *Saccharomyces cerevisiae* starting at 5% ethanol, with pronounced inhibitory effects observed from 7% onwards. These data confirm that *Y. lipolytica* is also sensitive to ethanol, reinforcing the notion that ethanol acts as a well-established growth inhibitor across various yeast species [14]. Nevertheless, *Y. lipolytica* exhibits a certain degree of tolerance to ethanolic stress, which may be linked to the activation of stress-response genes, particularly those involved in oxidative stress pathways, even under aerobic conditions. In *S. cerevisiae*, the transcription factor HAP1, which is involved in regulating respiration and limiting oxidative damage, plays a central role in the response to ethanol stress. Similar mechanisms may also be activated in *Yarrowia lipolytica* [12,15]. While the inhibitory mechanism observed at 7% ethanol was attributed to metabolic impairment, this interpretation is based on indirect physiological evidence such as reduced biomass formation, lower substrate consumption, and pronounced morphological alterations. Further studies incorporating cellular-level analyses—such as PI staining to assess membrane integrity and DCFH-DA assays to quantify ROS accumulation—will be necessary to clarify the specific mechanisms underlying ethanol-induced stress in *Y. lipolytica*. In addition to its moderate ethanol tolerance, *Y. lipolytica* demonstrates a uniquely high capacity for microbial oil production. In OMW, the lipid content reaches 68.28%, which is substantially higher than the modest increases reported for other oleaginous yeasts such as *Rhodotorula toruloides* and *Cryptococcus curvatus*, which typically exhibit only around a 20% enhancement under moderate ethanol stress [12]. This remarkable lipid accumulation highlights the exceptional metabolic robustness of *Y. lipolytica*. It underscores its potential advantage for industrial applications requiring high-yield microbial oil production under inhibitory or ethanol-containing conditions.

Finally, ethanol-induced stress also led to clear morphological changes in *Y. lipolytica* cells. Microscopic observations revealed increased cell size, altered cell shape, and accumulation of intracellular lipid droplets. These adaptations likely reflect metabolic adjustments and protective responses to maintain cellular integrity, which became more pronounced at higher ethanol concentrations, further supporting the view that *Y. lipolytica* actively remodels its physiology to cope with environmental stress.

### 4.2. Morphological Transitions and Their Implications

*Y. lipolytica* exhibits a characteristic ovoid morphology under optimal growth conditions, but environmental stress can trigger profound morphological transitions reflecting adaptive cellular remodeling. In our study, cells maintained a regular ovoid, well-dispersed morphology in the absence of ethanol (0%), confirming that neither olive mill effluent nor waste frying oil imposed morphological stress under normal conditions. However, ethanol exposure induced progressive and concentration-dependent alterations. At 5% ethanol, the appearance of cellular aggregates and filamentous structures suggests the activation of a stress-response program linked to cytoskeletal reorganization and altered cell wall dynamics.

Ethanol is known to intercalate into lipid bilayers, increasing plasma membrane fluidity and permeability, which disrupts ionic gradients and membrane-bound enzyme activities [24]. This membrane destabilization likely initiates a signaling cascade involving MAPK (mitogen-activated protein kinase) pathways and the reorganization of actin filaments, both of which are central to morphological plasticity in *Y. lipolytica* [25]. Under moderate stress, these pathways promote filamentous growth as a survival strategy, facilitating nutrient scavenging and surface adherence. Under higher ethanol concentrations, however, sustained oxidative and osmotic stress leads to cytoskeletal collapse and cell disorganization, compromising viability.

These findings align with previous observations by Bouchedja et al. [13] and Timoumi et al. [26], while expanding the interpretation toward the underlying physicochemical and molecular mechanisms. The observed transition from ovoid to filamentous forms therefore represents not only a morphological change but also a broader physiological shift orchestrated by ethanol-induced perturbations in membrane integrity, energy metabolism, and stress-responsive signaling networks.

### 4.3. Lipase Activity

This section presents the first investigation into the impact of ethanol-induced stress on lipase production by *Y. lipolytica*, a topic that remains largely unexplored in the literature. The objective was to evaluate how ethanol stress modulates enzymatic expression, particularly under different carbon source conditions, and to compare the findings with those reported under non-stressed conditions.

Significant differences were observed in the kinetics of lipase production by *Y. lipolytica* depending on the carbon source. In media containing spent frying oil, the peak of enzymatic activity occurred later, at 72 h, and reached a lower level (0.33 U/mL). In contrast, lipase activity in the OMW-based medium peaked earlier, at 48 h, with a higher value of 0.55 U/mL. These findings are consistent with those reported by [27], who observed an early induction of lipase synthesis in OMW-containing media using two different *Y. lipolytica* strains (W29 and IMUFRJ 50682). Similarly, *Y. lipolytica* W29 cultivated on used cooking oil exhibited a delayed peak in enzymatic activity at 72 h, as reported by [5,6]. The higher lipase activity in OMW can be attributed to the initial substrate composition: OMW contains a higher proportion of unsaturated fatty acids (C18:1, C18:2) and phenolic compounds, which may act both as readily hydrolysable substrates and as inducers of lipase gene expression through metabolic signaling. In contrast, WFO, richer in saturated fatty acids and poorer in phenolics, provides fewer cues for enzyme induction, resulting in lower lipase activity.

The temporal and quantitative differences observed between the two substrates may be attributed to their distinct chemical compositions. OMW, a by-product of olive oil extraction, is rich in complex triglycerols and phenolic compounds, which may induce lipase gene expression as a protective response to potentially toxic components. Furthermore, the study by Colacicco et al. [5] highlights that the nature of the carbon source plays a critical role in modulating both the level and kinetics of enzyme production by *Y. lipolytica*.

*Y. lipolytica* exhibited a biphasic response pattern, characteristic of cellular stress reactions, when exposed to varying ethanol concentrations (0%, 3%, 5%, and 7% *v*/*v*). The impact of ethanol also varied depending on the carbon source. Moderate ethanol concentrations (3–5%) led to a significant increase in lipase production in both media, suggesting the activation of adaptive cellular mechanisms. This enhancement may be reinforced in OMW cultures by the synergistic effect of unsaturated fatty acids and phenolic antioxidants, which facilitate membrane fluidity and stress resilience, supporting higher enzyme secretion. However, a marked decline in enzymatic activity was observed at 7% ethanol, indicating the existence of a critical threshold beyond which ethanol impairs metabolic function.

This reaction is consistent with previous research on other stressors, including hyperbaric stress. According to [28], raising air pressure to 5–8 bar increased oxygen transfer rates (OTR), boosting *Y. lipolytica*’s synthesis of lipase while simultaneously inducing an antioxidant response by activating protective enzymes such as superoxide dismutase. Under mild ethanol stress, similar metabolic defense mechanisms seem to be activated. By inducing adaptive responses such membrane remodeling and antioxidant pathway activation, ethanol may function as a moderate oxidative stress inducer at intermediate doses, temporarily increasing enzymatic productivity [29]. At higher ethanol concentrations (7% *v*/*v*), however, cells appear to reach a critical stress tolerance threshold beyond which their resilience is overwhelmed. A sharp decline in metabolic and enzymatic activity reflects this. The observed inhibition could be attributed to increased membrane damage, disruption of intracellular redox balance, or excessive accumulation of reactive oxygen species (ROS). Such cellular shock may lead to partial or complete cell lysis, a phenomenon previously reported in *P. pastoris* during secretory production of recombinant insulin under methanol induction. In that case, high methanol concentrations resulted in substantial cell lysis, highlighting the physiological limits of microbial hosts under intense stress, particularly when the target protein is secreted into the extracellular medium [11,30].

From a biotechnological perspective, the results highlight ethanol’s dual role as both an inducer and an inhibitor, depending on its concentration. Controlled ethanol exposure, combined with a substrate such as OMW, rich in unsaturated lipids and antioxidants, could be strategically exploited to enhance lipase yields. The use of low-cost, renewable substrates such as WFO and OMW further enhances the economic and environmental feasibility of the process [31]. The nearly twofold increase in lipase productivity under moderate ethanol stress demonstrates the exceptional adaptive plasticity of *Y. lipolytica*, reinforcing its potential as a robust host for sustainable enzyme production under stress-induced bioprocesses. The nearly twofold increase in lipase productivity under moderate ethanol stress in OMW demonstrates the exceptional adaptive plasticity of *Y. lipolytica*, emphasizing the crucial influence of substrate composition on enzyme production.

### 4.4. Fatty Acid Composition of Yeast Lipids

This work represents a first attempt to explore the impact of ethanol-induced stress on the lipid composition of microbial oils produced by *Y. lipolytica*.

Lipid accumulation in oleaginous microorganisms is primarily influenced by their intrinsic physiology, nutrient limitations, and environmental conditions [32]. In this study, nitrogen limitation combined with two distinct carbon sources and the application of ethanol stress were used to investigate the metabolic plasticity of *Y. lipolytica*.

Our results demonstrate that *Y. lipolytica* exhibits remarkable adaptability to ethanol-induced stress, reflecting considerable flexibility in its lipid metabolism. Under non-stress conditions, the fatty acid profiles were comparable across the two tested substrates (OMW and WFO) and were dominated by C16 and C18 fatty acids, including saturated (SFAs), monounsaturated (MUFAs), and polyunsaturated (PUFAs) species. However, the differences observed mainly concern the quantitative profile of fatty acids, while the total lipid yields remained comparable between the two carbon sources. This suggests that the substrate influences fatty acid composition rather than overall lipid productivity [8,33]. Similar findings have been reported by [34] who observed that the fatty acid composition of intracellular lipids in yeasts cultivated on lipid-rich substrates closely reflects the composition of the growth medium. Such substrate-dependent modulation likely arises from selective channeling of exogenous lipids into the ex novo synthesis pathway and from transcriptional regulation of key enzymes such as acyl-CoA oxidases, desaturases, and elongases.

Upon exposure to ethanol stress, a marked increase in MUFAs—particularly oleic acid (C18:1)—was observed in both culture media. This shift likely reflects an adaptive upregulation of Δ9-desaturase, the key enzyme that catalyzes the conversion of stearic acid (C18:0) to oleic acid. This enzymatic adjustment serves to counteract the rigidifying effect of ethanol on the plasma membrane, since increased MUFA content enhances fluidity and prevents phase transition of membrane lipids. Although this interpretation is inferred from fatty acid composition and was not supported by direct enzymatic or gene expression evidence, lipid remodeling is consistent with stress-adaptive mechanisms previously described in *R. toruloides*, in which combined stress factors (ethanol, elevated temperature, and osmotic pressure) stimulate unsaturated fatty acid accumulation to preserve membrane fluidity. In *Y. lipolytica*, the dominance of oleic acid (C18:1) under all conditions suggests a finely regulated desaturation process, potentially involving redox-sensitive transcription factors that modulate lipid biosynthetic genes under oxidative or ethanol stress [12]. Moreover, the predominance of oleic acid (C18:1) in microbial oils under all culture conditions may result not only from Δ9-desaturase activity but also from the strain’s selective uptake and utilization of oleic acid through the ex novo lipid accumulation pathway. In response to oxidative or ethanol stress, an increased proportion of unsaturated fatty acids (C18:1, C18:2) was observed, supporting their role in enhancing membrane fluidity and stress resilience [12,35,36]. This response parallels observations in other oleaginous yeasts, where unsaturated lipid enrichment minimizes ROS-induced lipid peroxidation and stabilizes membrane-bound proteins.

The influence of the initial substrate composition was particularly evident when comparing OMW and WFO. OMW, which initially contained a higher proportion of unsaturated fatty acids (ΣMUFA ≈ 44.7%; ΣPUFA ≈ 37.6%) compared to WFO (ΣMUFA ≈ 50.5%; ΣPUFA ≈ 23.4%; ΣSFA ≈ 26.1%), favored the synthesis of more unsaturated microbial lipids. This trend indicates that yeast partially mirrors the substrate lipid profile by selectively incorporating and metabolizing exogenous fatty acids. The dynamics of PUFA accumulation appeared substrate-dependent. In cultures grown on OMW, PUFA levels remained relatively stable even under high ethanol stress (7%), possibly due to the presence of polyphenols with antioxidant properties, which may limit lipid peroxidation. Additionally, the activation of endogenous antioxidant systems such as superoxide dismutases and catalases could contribute to this stability [10,12,37]. Conversely, in cultures grown on waste frying oil, a biphasic response was observed: PUFA levels transiently increased under moderate ethanol stress (5%), followed by a significant decline at higher ethanol concentrations (≥7%). This pattern suggests an initial protective activation of desaturases followed by oxidative damage when ROS accumulation exceeds antioxidant capacity. This decrease likely reflects enhanced lipid peroxidation caused by excessive production of reactive oxygen species (ROS) under stressful conditions [10,12,38]. The interplay between ROS production, antioxidant defenses, and lipid remodeling thus defines the tolerance threshold of *Y. lipolytica* under ethanol exposure.

On OMW—a lipid-rich substrate likely dominated by triglycerols—the SFA content decreased progressively from 20.04% to approximately 14%. This reduction indicates enhanced fatty acid desaturation, a well-documented adaptive mechanism to preserve membrane fluidity under stress, presumably catalyzed by membrane-bound desaturases [12]. Such regulation is energetically demanding and relies on NADPH availability, linking lipid remodeling to central carbon metabolism and redox balance. In contrast, in cultures grown on WFO, SFA levels remained relatively stable (~8%), suggesting either a pre-existing saturation of the medium due to limited availability of unsaturable substrates or substrate-specific metabolic regulation influenced by the initial composition of the oil. This finding aligns with the initial composition of WFO, characterized by higher levels of saturated (C16:0, C18:0) and lower levels of polyunsaturated fatty acids, which could restrict the desaturation potential of the yeast during adaptation to ethanol stress. Therefore, the chemical nature of the substrate—its fatty acid composition and redox-active compounds—plays a crucial role in determining the direction of lipid remodeling. OMW, rich in oleic and linoleic acids and containing phenolic antioxidants, provides a more favorable environment for unsaturated lipid synthesis and stabilization. At the same time, WFO promotes the production of more saturated, oxidation-resistant microbial oils.

Notably, very-long-chain fatty acids (C20:1 and C20:2) emerged exclusively in cultures on OMW-based medium exposed to moderate ethanol stress (3%). In addition, differentiating fatty acids such as linoleic acid (C18:2) were likely generated through transformation of the initial lipid profile, dominated by oleic acid, via intracellular elongation and desaturation pathways in *Y. lipolytica*. The transient synthesis of these long-chain and polyunsaturated species indicates the activation of lipid elongase and desaturase complexes, possibly as part of a stress-induced membrane remodeling response aimed at optimizing fluidity and permeability, and may be triggered by OMW’s chemical complexity, including phenolic compounds, and the oxidative stress induced by ethanol exposure [8,28]. This behavior reinforces the idea that substrate composition—particularly the redox-active compounds in OMW—modulates the timing and extent of lipid remodeling under ethanol stress.

Together, these observations reveal complex cross-talk among environmental stress, redox homeostasis, and lipid biosynthesis pathways, underscoring the metabolic versatility of *Y. lipolytica* as an oleaginous yeast [12,39].

These findings highlight that the ethanol-induced remodeling of fatty acids in *Y. lipolytica* is not merely a biochemical adaptation but a determinant of the oxidative stability and functional quality of the resulting microbial oils, aspects further examined in the following section.

### 4.5. Insights into Oxidative Stability of Microbial Oils

The oxidative stability of microbial oils produced by *Yarrowia lipolytica* appears to be closely related to both the nature of the carbon substrate and the level of ethanol stress applied during cultivation. Under these conditions, modulation of the lipid composition—particularly variations in the relative proportions of saturated (SFA), monounsaturated (MUFA), and polyunsaturated fatty acids (PUFA)—plays a key role in determining the oils’ resistance to thermal oxidation [40,41].

A high MUFA content, especially oleic acid (C18:1), combined with a moderate proportion of long-chain fatty acids, constitutes a favorable factor for thermal stability. Conversely, an excess of PUFA—highly prone to oxidation due to their multiple double bonds—tends to reduce this stability significantly. Literature data confirm that lipid profiles dominated by PUFA, such as in fish oils, are more susceptible to oxidation due to the rapid formation of unstable hydroperoxides [40,42,43]. Furthermore, the intrinsic richness in antioxidant compounds such as polyphenols, present in certain substrates like olive mill wastewater (OMW), may also influence the oxidative resistance of microbial oils. However, the protective effects of polyphenols remain controversial, with some studies suggesting a limited effect on thermal properties, which are largely dependent on the type of oil, polyphenol concentration, and the analytical method used [40].

In this study, the oils extracted from *Y. lipolytica* grown on OMW exhibited enhanced oxidative stability compared to those obtained from WFO. This behavior can be attributed to their higher proportion of monounsaturated fatty acids and the presence of phenolic residues derived from the growth medium. The coexistence of unsaturated, oxidation-resistant MUFAs (mainly oleic acid) and natural antioxidants may act synergistically to delay lipid peroxidation initiation. Conversely, oils from WFO-based cultures, characterized by higher saturation and lower antioxidant potential, displayed reduced adaptability to ethanol stress and were more susceptible to oxidative degradation. These results reinforce the idea that both substrate-derived compounds and metabolic stress define the oxidative robustness of microbial oils.

It is also likely that ethanol stress exerts a dual effect: on one hand, by redirecting the yeast’s lipid metabolism toward more or less stabilizing profiles, and on the other, by inducing oxidative cellular conditions that may impair the quality of the lipids produced under high ethanol concentrations. This duality highlights the importance of maintaining redox balance during cultivation, as excessive accumulation of reactive oxygen species (ROS) can trigger lipid peroxidation and compromise oil integrity. In contrast, moderate stress can stimulate adaptive responses that enhance resistance to oxidation.

Altogether, these findings indicate that the oxidative stability of *Y. lipolytica* oils is governed by the delicate interplay between fatty acid composition, residual antioxidant content, and the intensity of environmental stress. Optimizing culture parameters—particularly substrate selection, nitrogen availability, and ethanol concentration—thus represents a strategic approach to tailor lipid profiles and improve the functional stability of microbial oils. This strategy offers promising avenues for developing biotechnological lipids with controlled oxidative properties, suitable for applications in food, cosmetics, and biolubricant formulations where oxidative resistance is a critical quality criterion.

## 5. Conclusions

This study provides the first evidence of ethanol-induced stress affecting both lipase production and lipid remodeling in *Y. lipolytica* cultivated on hydrophobic waste-derived substrates. Moderate ethanol stress significantly enhanced lipase activity and altered fatty acid composition, highlighting the yeast’s strong metabolic adaptability. These findings support the potential of *Y. lipolytica* as a microbial platform for sustainable co-production of enzymes and lipids from agro-industrial by-products.

Nevertheless, this work was limited to shake-flask experiments using a single wild-type strain (L2). Future studies should focus on testing other *Y. lipolytica* strains (including reference and engineered strains) to assess strain-dependent variability, as well as on bioreactor-scale validation and metabolic engineering strategies to enhance ethanol tolerance and process scalability toward industrial applications.

## Figures and Tables

**Table 1:** Fatty acid composition of waste frying oil (WFO) and olive mill wastewater (OMW). Values are mean ± SD and expressed as % of total fatty acids. SFA—saturated fatty acids; MUFA—monounsaturated fatty acids; PUFA—polyunsaturated fatty acids.

Fatty Acid	WFO	OMW
C16:0 (Palmitic acid)	11.71 ± 0.54	13.83 ± 0.71
C16:1 (Palmitoleic acid)	00.00 ± 0.00	0.97 ± 0.05
C18:0 (Stearic acid)	14.44 ± 1.61	3.95 ± 0.06
C18:1 (Oleic acid)	50.45 ± 2.43	43.70 ± 0.25
C18:2 (Linoleic acid)	19.99 ± 4.04	33.35 ± 0.37
C18:3 (α-Linolenic acid)	3.42 ± 0.54	4.22 ± 0.10
Σ_SFA_	26.15 ± 1.07	17.78 ± 0.66
Σ_MUFA_	50.45 ± 2.43	44.66 ± 0.20
Σ_PUFA_	23.41 ± 3.50	37.57 ± 0.47

**Table 2:** Fatty acid composition of microbial oil from *Y. lipolytica* L2 (KF156787) grown in a WFO medium under ethanol stress. Values are mean ± SD and expressed as % of total fatty acids. SFA—saturated fatty acids; MUFA—monounsaturated fatty acids; PUFA—polyunsaturated fatty acids. “Other” is the sum of remaining identified fatty acids. The values with the same uppercase letters (A–D) within the row did not differ significantly (Tukey’s HSD test, α = 0.05).

Fatty Acid	Ethanol in Medium (% *v*/*v*)			
	0	3	5	7
C16:0 (Palmitic acid)	5.54 ± 1.21 ^A^	6.16 ± 0.09 ^A^	5.85 ± 0.03 ^A^	6.00 ± 0.05 ^A^
C16:1 (Palmitoleic acid)	1.95 ± 0.46 ^A^	0.76 ± 0.01 ^B^	0.60 ± 0.00 ^B^	0.42 ± 0.00 ^B^
C18:0 (Stearic acid)	2.20 ± 0.01 ^C^	2.62 ± 0.06 ^A^	2.39 ± 0.00 ^B^	2.45 ± 0.08 ^B^
C18:1 (Oleic acid)	38.67 ± 1.59 ^D^	52.58 ± 1.04 ^C^	56.16 ± 0.53 ^B^	61.49 ± 0.19 ^A^
C18:2 (Linoleic acid)	19.44 ± 0.89 ^C^	23.42 ± 0.42 ^B^	26.27 ± 0.05 ^A^	23.69 ± 0.01 ^B^
C18:3 (α-Linolenic acid)	4.62 ± 0.19 ^D^	6.71 ± 0.13 ^B^	7.01 ± 0.02 ^A^	5.12 ± 0.07 ^C^
C20:1 (Eicosenoic acid)	16.25 ± 2.50 ^A^	4.25 ± 0.11 ^B^	0.86 ± 0.01 ^C^	0.03 ± 0.03 ^C^
Other	11.35 ± 1.87 ^A^	3.53 ± 0.21 ^B^	0.87 ± 0.58 ^C^	0.83 ± 0.09 ^C^
Σ_SFA_	7.74 ± 1.22 ^A^	8.77 ± 0.16 ^A^	8.24 ± 0.03 ^A^	8.44 ± 0.03 ^A^
Σ_MUFA_	56.86 ± 0.45 ^B^	57.58 ± 0.92 ^B^	57.61 ± 0.54 ^B^	61.94 ± 0.16 ^A^
Σ_PUFA_	24.06 ± 1.08 ^D^	30.13 ± 0.54 ^B^	33.27 ± 0.03 ^A^	28.81 ± 0.06 ^C^

**Table 3:** Fatty acid composition of microbial oil from *Y. lipolytica* L2 (KF156787) grown in a OMW medium under ethanol stress. Values are mean ± SD and expressed as % of total fatty acids. SFA—saturated fatty acids; MUFA—monounsaturated fatty acids; PUFA—polyunsaturated fatty acids. The values with the same uppercase letters (A–C) within the row did not differ significantly (Tukey’s HSD test, α = 0.05).

Fatty Acid	Ethanol in Medium (% *v*/*v*)			
	0	3	5	7
C16:0 (Palmitic acid)	16.00 ± 2.50 ^A^	9.77 ± 0.21 ^B^	11.74 ± 0.11 ^B^	10.94 ± 0.04 ^B^
C16:1 (Palmitoleic acid)	3.07 ± 0.33 ^A^	0.00 ± 0.00 ^B^	0.00 ± 0.00 ^B^	0.00 ± 0.00 ^B^
C18:0 (Stearic acid)	4.05 ± 0.39 ^A^	3.31 ± 0.01 ^C^	3.79 ± 0.01 ^AB^	3.47 ± 0.01 ^BC^
C18:1 (Oleic acid)	40.56 ± 1.44 ^B^	41.70 ± 0.18 ^B^	47.33 ± 0.07 ^A^	48.61 ± 0.20 ^A^
C18:2 (Linoleic acid)	32.55 ± 0.78 ^A^	26.77 ± 0.34 ^C^	32.30 ± 0.06 ^A^	31.06 ± 0.01 ^B^
C18:3 (α-Linolenic acid)	3.80 ± 0.22 ^B^	3.79 ± 0.04 ^B^	3.98 ± 0.03 ^B^	4.35 ± 0.13 ^A^
C20:1 (Eicosenoic acid)	0.00 ± 0.00 ^C^	8.36 ± 0.42 ^A^	1.58 ± 0.12 ^B^	1.58 ± 0.12 ^B^
C20:2 (Eicosadienoic acid)	0.00 ± 0.00 ^B^	6.31 ± 0.34 ^A^	0.00 ± 0.00 ^B^	0.00 ± 0.00 ^B^
Σ_SFA_	20.04 ± 2.11 ^A^	13.08 ± 0.21 ^C^	15.52 ± 0.11 ^B^	14.41 ± 0.05 ^BC^
Σ_MUFA_	43.62 ± 1.10 ^C^	50.06 ± 0.24 ^A^	48.21 ± 0.08 ^B^	50.19 ± 0.08 ^A^
Σ_PUFA_	36.34 ± 1.00 ^AB^	36.87 ± 0.04 ^A^	36.28 ± 0.04 ^AB^	35.41 ± 0.13 ^B^

**Table 4:** Oxidative-stability parameters of microbial oil from *Y. lipolytica* L2 (KF156787) grown on waste frying oil, measured by pressure differential scanning calorimetry (PDSC) at 120 °C. τ_on_—oxidation onset time; τ_max_—time to maximum oxidation rate. Values are mean ± SD. The values with the same uppercase letters (A–B) within the column did not differ significantly (Tukey’s HSD test, α = 0.05).

Ethanol Addition (*v*/*v* %)	τ_on_ (min)	τ_max_ (min)
0	1.02 ± 0.18 ^A^	3.45 ± 0.13 ^B^
3	0.86 ± 0.45 ^AB^	5.02 ± 0.65 ^A^
5	0.50 ± 0.18 ^B^	4.51 ± 0.37 ^A^
7	0.97 ± 0.23 ^AB^	3.35 ± 0.67 ^B^

**Table 5:** Oxidative-stability parameters of microbial oil from *Y. lipolytica* L2 (KF156787) grown on olive mill wastewater, measured by pressure differential scanning calorimetry (PDSC) at 120 °C. τ_on_—oxidation onset time; τ_max_—time to maximum oxidation rate. Values are mean ± SD. The values with the same uppercase letters (A–D) within the column did not differ significantly (Tukey’s HSD test, α = 0.05).

Ethanol Addition (*v*/*v* %)	τ_on_ (min)	τ_max_ (min)
0	30.48 ± 0.80 ^B^	35.73 ± 0.62 ^B^
3	47.07 ± 3.92 ^A^	54.04 ± 1.99 ^A^
5	12.15 ± 0.12 ^C^	16.92 ± 0.16 ^C^
7	4.99 ± 0.21 ^D^	9.10 ± 0.37 ^D^

## Data Availability

The original contributions presented in this study are included in the article. Further inquiries can be directed to the corresponding author.

## Abbreviations

The following abbreviations are used in this manuscript:

ANOVA	Analysis of variance
DCW	Dry Cell Weight
DSC	Differential Scanning Calorimetry
FA	Fatty Acid
FAME	Fatty Acid Methyl Ester
FID	Flame Ionization Detector
GC	Gas Chromatography
HAP1	Heme-Activated Protein 1
ISO	International Organization for Standardization
MUFA	Monounsaturated Fatty Acid
OMW	Olive Mill Wastewater
OTR	Oxygen Transfer Rate
PDSC	Pressure Differential Scanning Calorimetry
PUFA	Polyunsaturated Fatty Acid
ROS	Reactive Oxygen Species
SFA	Saturated Fatty Acid
SD	Standard Deviation
WFO	Waste Frying Oil
